# SpArking change for patients with psoriatic arthritis and axial spondyloarthritis in the UK: results from a UK Delphi consensus study

**DOI:** 10.1093/rap/rkag005

**Published:** 2026-01-09

**Authors:** Philip S Helliwell, Antoni Chan, David Crosbie, Pauline Ho, Elena Nikiphorou, Andrew Pothecary, Raj Sengupta, Ben Thompson

**Affiliations:** Leeds Institute of Rheumatic and Musculoskeletal Medicine, University of Leeds, Leeds, UK; University Department of Rheumatology, Royal Berkshire NHS Foundation Trust, Reading, UK; Department of Rheumatology, Queen Elizabeth University Hospital, Glasgow, UK; Kellgren Centre for Rheumatology, Manchester University NHS Foundation Trust, Manchester, UK; Centre for Musculoskeletal Research, University of Manchester, Manchester, UK; Centre for Rheumatic Diseases, King’s College London, London, UK; Rheumatology Department, King’s College Hospital, London, UK; Rheumatology and Biologics Lead Pharmacist, Royal Cornwall Hospitals NHS Trust, Truro, UK; Royal National Hospital for Rheumatic Diseases, Royal United Hospitals, Bath, UK; Rheumatology Department, The Newcastle-upon-Tyne Hospitals NHS Foundation Trust, Newcastle upon Tyne, UK

**Keywords:** psoriatic arthritis, axial spondyloarthritis, patient-centred care, Delphi technique, patient empowerment, self-management, multidisciplinary care team, United Kingdom

## Abstract

**Objectives:**

To establish a UK-specific consensus on improving standards of care for patients with PsA and axial SpA through patient empowerment, education, access and optimal treatment approaches.

**Methods:**

A modified Delphi methodology was employed. A steering group of UK rheumatologists and pharmacists developed 56 consensus statements across four domains: patient empowerment, patient and healthcare professional (HCP) knowledge, access to healthcare and treatment principles. These statements were tested with a panel of 100 UK rheumatologists using a 4-point Likert scale. Consensus was predefined as ≥75% agreement.

**Results:**

Consensus was achieved for 98% (55/56) of statements; 93% (52/56) reached strong consensus (≥90%). Statements supported embedding patient empowerment tools (e.g. patient-reported outcome measures, patient activation measures), implementing patient-initiated follow-up and ensuring shared decision-making. Respondents strongly endorsed multidisciplinary care, tailored educational resources, psychological support and timely access to physiotherapy and biologics. Treatment decisions should prioritise clinical need and patient goals rather than cost alone.

**Conclusion:**

This UK Delphi consensus highlights expert agreement that best practice care for PsA and axial SpA should centre on patient empowerment, supported by multidisciplinary teams, education and equitable access to treatments. Implementing personalised, holistic care models has the potential to improve patient outcomes and reduce healthcare burden. Further research should validate these recommendations with patients and explore strategies for their integration into National Health Service practice.

## Graphical abstract

**Figure:**
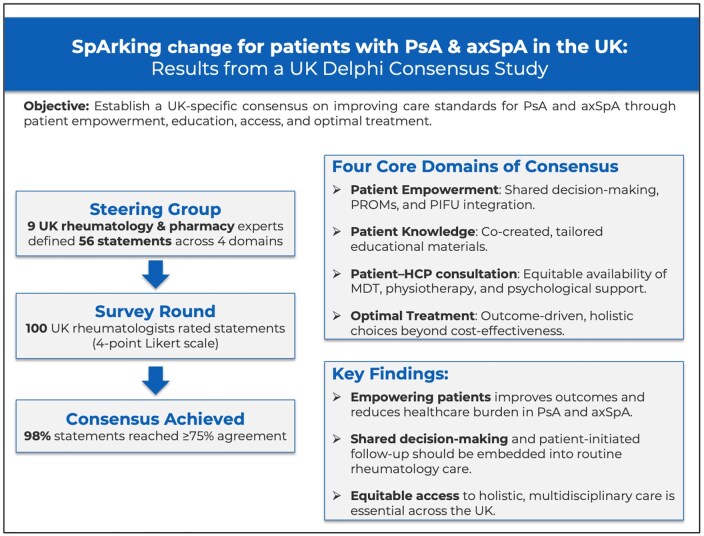

Key messages

- Empowering patients improves outcomes and reduces healthcare burden in PsA and axial SpA.
- Shared decision-making and patient-initiated follow-up should be embedded into routine rheumatology care.
- Equitable access to holistic, multidisciplinary care is essential across the UK.

## Introduction

PsA and axial SpA (axSpA) are inflammatory rheumatic diseases belonging to the SpA group. PsA primarily affects the joints and the skin of individuals with active or latent psoriasis. Extramusculoskeletal manifestations (EMMs) may also affect the nails and potentially the gut (IBD) or eyes (uveitis). PsA is also associated with cardiovascular, psychological and metabolic comorbidities [1].

AxSpA is a form of inflammatory arthritis of the axial skeleton but can also include peripheral (arthritis, enthesitis and dactylitis) and EMMs such as uveitis, IBD and psoriasis. AxSpA encompasses both radiographic (r-axSpA/AS) and non-radiographic (nr-axSpA) forms, distinguished by the presence or absence of radiographic sacroiliitis [2].

The burden of these chronic conditions goes beyond the physical impact of disease manifestations and both axSpA and PsA patients have been shown to experience a significantly reduced health-related quality of life compared with both normal values as well as individuals with psoriasis alone [3]. Depression is an important comorbidity and is associated with fatigue, not engaging in exercise and limited physical function [4]. As a consequence of both the physical and mental impacts of these conditions, patients experience lower employment rates and greater discrimination at work [5, 6].

Improving outcomes involves more than simply offering pharmacological treatment. As chronic conditions, a holistic approach to management is needed that includes patient empowerment and consideration of patient-reported outcomes. Analysis of almost 14 000 patients found that, compared with RA, PsA was associated with lower mean physician global assessment (18.6 *vs* 27.3), higher patient global assessment (43.2 *vs* 36.9), comparable pain (38.9 *vs* 39.5) and lower fatigue (41.1 *vs* 43.4) scores. However, patients with axSpA had comparable mean physician global assessment (25.5 *vs* 27.3) and higher patient global assessment (50.2 *vs* 36.9), pain (46.1 *vs* 39.5) and fatigue (48.3 *vs* 43.4) scores to those with RA [7]. These results suggest some disparity in the assessment of disease impact between patients and physicians.

Increasing numbers living for longer with chronic conditions continues to add demand to already overstretched healthcare services in the UK [8]. To improve standards of care for chronic conditions such as axSpA and PsA, patients should be empowered to manage their own care with appropriate support [9].

The 2021 EULAR recommendations provide a set of overarching principles and recommendations for self-management strategies, including the need for patient education, and the role of lifestyle advice, physical activity, emotional well-being and digital healthcare to support self-management and improve self-efficacy [10].

This work is designed to build on the Pan-European Rheumacensus [11] initiative centred around four key aims and objectives [12]: patient empowerment, patient knowledge, patient–healthcare professional consultation and optimal initial treatment.

On the basis that patient empowerment in PsA and axSpA is currently suboptimal, the Rheumacensus [11, 12] initiative identified a number of ‘calls to action’ intended to elevate the standard of care (SoC) for axSpA and PsA.

While EULAR [1, 2] recommendations provide comprehensive frameworks for clinical management, they are not designed to account for the structural, organisational and commissioning characteristics of the UK healthcare system. The National Health Service (NHS) is a publicly funded, regionally devolved service in which the planning and delivery of rheumatology care is influenced by integrated care systems (ICSs) that set local priorities and budgets [13]. Consequently, access to multidisciplinary services, physiotherapy, psychological support and advanced therapies varies significantly across regions, as evidenced by recent national audits and GIRFT findings [14, 15]. Developing a UK-specific consensus thus enables alignment of best practice recommendations with the realities of NHS delivery models, ensuring that principles of patient empowerment, equity and holistic care can be operationalised consistently within the UK context. The current UK-specific modified Delphi consensus is intended to provide a UK-specific perspective on how to improve the SoC in PsA and axSpA through patient empowerment and a multidisciplinary approach that considers the patient’s personal goals for treatment. The findings are intended to align with and extend ongoing EULAR [10] implementation efforts aimed at embedding self-management and patient empowerment within routine rheumatology care. By translating these international principles into recommendations tailored to the NHS structure, this work is intended to support the broader European objective of harmonising high-quality, patient-centred care across diverse healthcare systems.

## Methods

**Figure 1:**
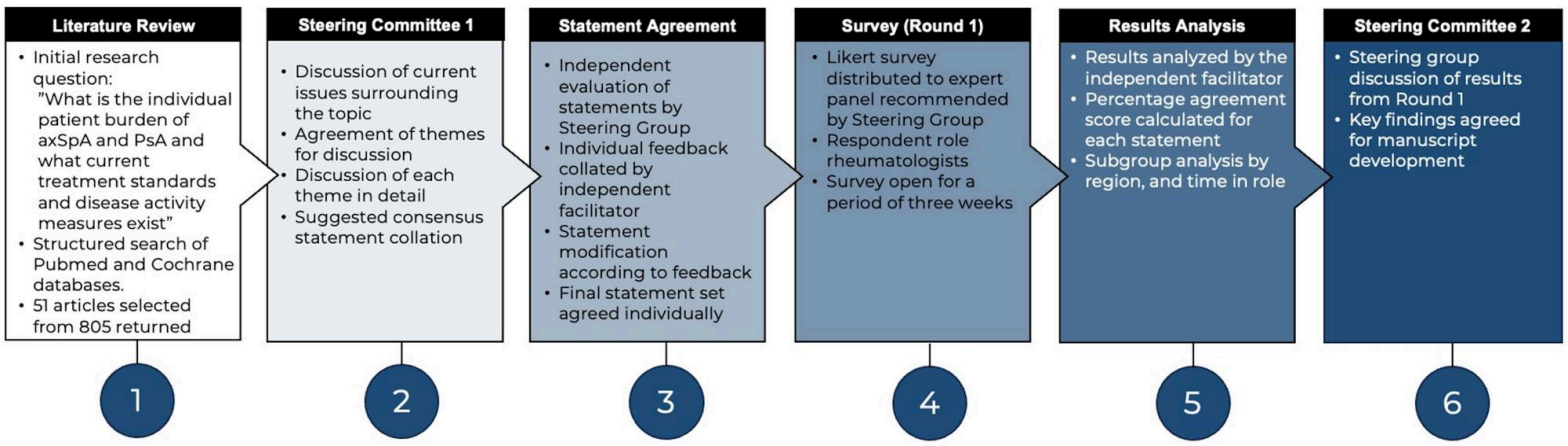
Modified Delphi study design

The process followed a modified Delphi methodology (Fig. 1). In June 2024, a literature review of best practices in axSpA and PsA management was performed using the PubMed and Cochrane databases along with a general web search.

The initial research question set for the purposes of the literature review was: What is the individual patient burden of axSpA and PsA and what current treatment standards and disease activity measures exist? The search terms used included ‘axial spondyloarthritis’, ‘psoriatic arthritis’ and ‘patient empowerment’ and searches were restricted to publications since 2015 that were available as full text. A total of 805 articles were identified and, after manual assessment, 51 were included. The findings were used to build a further set of questions to underpin the agenda of a steering group meeting held in July 2024.

The independent facilitator (Triducive Partners Ltd, UK) convened a steering group of UK-based healthcare professionals (HCPs). The group included seven consultant rheumatologists, one rheumatology specialist pharmacist and one chief pharmacist; selection was based on published research (including international guidelines) and experience in treating axSpA and PsA.

Four main domains of focus were agreed upon: patient empowerment in PsA and axSpA, patient and HCP knowledge in PsA and axSpA, access to healthcare and principles of optimal treatment. These themes were discussed and 63 statements were developed by the steering group. The statements were then sent to the individual members of the steering group for independent review. The steering group received a copy of the agreed upon statements for review and, over a 2-week period, the individual members provided written feedback to the facilitator. The facilitator updated the statements in line with the feedback received and the edited set was sent to the group for agreement prior to the survey. The survey was piloted with 10 respondents prior to wider distribution; this was to ensure that the survey functioned correctly—no feedback was sought from respondents in the pilot. The resulting 56 statements (Table 1) were developed into a survey for distribution to a wider panel.

**Table 1:** Overall agreement scores and percent response for each agreement level.

No.	Statement	Strongly agree	Agree	Disagree	Strongly disagree	Overall agreement
Domain A: Patient empowerment in PsA and axSpA						
1	PsA and axSpA patients (and their symptoms) should be listened to by all HCPs involved in the diagnosis and care and not ignored	79%	20%	0%	1%	99%
2	PsA and axSpA patients should be made aware of their role and rights in their care and supported to voice their experience, ask questions and state their treatment goals	79%	21%	0%	0%	100%
3	HCPs should create a permissive care environment (i.e. through open, free-flowing conversation) so PsA and axSpA patients feel confident and comfortable discussing their needs	76%	23%	1%	0%	99%
4	The visibility and engagement of patients and patient advocacy groups (PAGs) should be improved to enhance patient support and strengthen the patient voice at an organisational level	49%	48%	3%	0%	97%
5	PsA and axSpA patients should receive individually tailored educational support to help them understand how best to live with a chronic condition	69%	28%	3%	0%	97%
6	Appropriate PsA and axSpA patients (e.g. those with comorbidities such as depression and anxiety) should be offered psychological support to help cope with life with a chronic condition	70%	30%	0%	0%	100%
7	Currently available patient empowerment measures should be audited and tailored to individual patient needs before implementation in the clinic	43%	51%	6%	0%	94%
8	PsA and axSpA patients should be informed and involved in shared decision-making for their treatment decisions as this can improve outcomes, with HCPs providing the necessary information and support in order for them to do so	78%	21%	1%	0%	99%
9	PsA and axSpA patients should be encouraged and supported to self-manage (i.e. through PAMs), as this can improve outcomes	71%	27%	2%	0%	98%
10	PsA and axSpA patients should be able to discuss their PROMs with their HCP	64%	34%	2%	0%	98%
11	An MDT approach should be implemented to ensure the best possible outcomes for PsA and axSpA patients	77%	19%	3%	1%	96%
12	Outcomes may improve if patients know how and why to engage in structured follow-up and monitoring (e.g. through PIFU)	70%	29%	1%	0%	99%
Domain B: Patient knowledge in PsA and axSpA						
13	PsA and axSpA patients should be directed to high-quality, validated, easy-to-access educational material	76%	23%	1%	0%	99%
14	PsA and axSpA patients should be provided with bite-sized, interactive educational resources tailored to their individual needs, covering any aspect of living with axSpA or PsA that interests them (including clinical, holistic, and health economic topics) by their clinician	55%	43%	2%	0%	98%
15	Reliable sources of information are important to enable patients to understand treatment options and prognosis	80%	19%	1%	0%	99%
16	Reliable sources of information for patients presenting with PsA or axSpA symptoms should be educationally appropriate for patients at all levels of understanding	79%	20%	1%	0%	99%
17	Reliable sources of information for patients presenting with PsA or axSpA symptoms should be culturally appropriate for patients from all backgrounds	73%	25%	1%	1%	98%
18	Reliable sources of information for patients presenting with PsA or axSpA symptoms should be accessible to people without relying solely on digital media or devices	74%	24%	2%	0%	98%
19	Collaboration to develop tailored, holistic educational materials with patients, covering the full patient journey from diagnosis through all life stages, as well as information on comorbidities, is needed	66%	30%	4%	0%	96%
20	HCPs and PAGs should collaborate to co-create, validate and distribute high-quality information to PsA and axSpA patients, becoming recognised sources of reliable and accessible information	57%	42%	1%	0%	99%
21	Patient decision aids should be developed and validated with patient input to help them make appropriate decisions	56%	41%	3%	0%	97%
22	NASS resources can be used to support axSpA patients and should be routinely promoted to patients and HCPs	74%	25%	1%	0%	99%
23	Such information needs strong visibility and clear signposting	73%	26%	1%	0%	99%
24	PsA and axSpA patients tend to be more motivated by their ability to achieve life goals than by the achievement of specific medical targets	51%	44%	5%	0%	95%
25	Patient health priorities should be sought and then inform the choice of treatment disease management (both non-pharmacological and pharmacological)	65%	35%	0%	0%	100%
26	When setting treatment targets for PsA and axSpA patients, their goals should be understood and accommodated	69%	30%	1%	0%	99%
27	Broader education, beyond the patient, is necessary to improve knowledge of PsA and axSpA among primary care, dermatologists and the general public	56%	44%	0%	0%	100%
28	Allied health professionals should be educated to better identify and refer PsA and axSpA	60%	37%	2%	1%	97%
29	Primary care professionals should be educated to better identify and refer PsA and axSpA	69%	29%	2%	0%	98%
30	Primary care has a critical role to play in identifying patients presenting with PsA and axSpA symptoms and referring them to rheumatology	75%	25%	0%	0%	100%
Domain C: Access to healthcare						
31	Varying levels of awareness and knowledge can negatively impact the outcomes of patients with PsA	48%	47%	5%	0%	95%
32	There is a need to develop and utilise new and existing tools to better prepare patients for consultations	39%	53%	8%	0%	92%
33	Different models of delivering patient care for PsA should be explored in the NHS	37%	52%	10%	1%	89%
34	HCP communication techniques, including active listening, motivational interviewing and soft skills, should be supported	61%	32%	6%	1%	93%
35	PsA patients who are experiencing a flare-up should have access to prompt, appropriately skilled help and advice, usually via their rheumatology team	77%	23%	0%	0%	100%
36	The correct use of PIFU ensures the appropriate patient is more likely to see the right HCP at the appropriate time	48%	45%	7%	0%	93%
37	Collaboration between the patient and HCPs (e.g. specialist rheumatology nurses and physiotherapists) is crucial to ascertain patients’ individual needs to inform and set tailored treatment goals	71%	26%	3%	0%	97%
38	It is important to actively listen to the patient’s lived experience of axSpA and take this into account rather than solely using laboratory results and clinical findings to guide management	69%	28%	3%	0%	97%
39	axSpA patients should be supported to be active partners with HCPs in decisions about their treatment	69%	29%	2%	0%	98%
40	Consultations should be structured and tailored as a collaboration between the HCP and patient to cover all aspects of axSpA management that are important to them to reach a shared treatment decision	61%	35%	4%	0%	96%
41	A personalised flare management plan should be made available to patients	50%	45%	5%	0%	95%
42	A personalised biologic management plan should be made available to patients	51%	43%	6%	0%	94%
Domain D: Optimal treatment						
43	Treatment choice for PsA should be based on clinical need rather than cost-effectiveness alone	45%	50%	4%	1%	95%
44	Treatment choice for PsA should be outcome-driven rather than prescriptive to guidelines	50%	43%	7%	0%	93%
45	It is important to adopt data, tools and algorithms to better understand drug comparisons and optimal treatments	58%	37%	5%	0%	95%
46	Routine outcome data would be useful to identify patients who may benefit from alternative first-line treatments	50%	43%	6%	1%	93%
47	Extra-musculoskeletal manifestations such as psoriasis, IBD and uveitis should be taken into consideration when deciding what medication options are possible	78%	21%	1%	0%	99%
48	A longer period from symptom onset is required to assess outcomes in PsA compared with RA	27%	47%	26%	0%	74%
49	ICSs should develop clinical guidelines based on the clinical needs of their PsA populations	44%	46%	9%	1%	90%
50	Payors/commissioners should engage with patients and patient organisations to improve the understanding of each other’s roles, perspectives and experiences	48%	46%	6%	0%	94%
51	The patient perspective should be included in formulary committees and guideline development	41%	48%	10%	1%	89%
52	Equity of timely access to biologics across regions should be ensured	66%	33%	1%	0%	99%
53	axSpA patients with comorbidities (e.g. hypertension, obesity, depression, IBD, uveitis) may receive suboptimal or delayed care	49%	38%	13%	0%	87%
54	Access to physiotherapy improves outcomes for patients with axSpA	76%	24%	0%	0%	100%
55	Hydrotherapy should be offered and easily accessible to patients with axSpA	59%	35%	4%	2%	94%
56	Psychological support should be offered and easily accessible to patients with axSpA	61%	39%	0%	0%	100%

Recruitment of panel members was via a third-party specialist provider (M3 Global Research, Fort Washington, PA, USA) according to the following criteria: rheumatologist within the UK and experience working in axSpA and/or PsA.

The survey presented each statement along with a 4-point Likert scale (‘strongly agree’, ‘tend to agree’, ‘tend to disagree’ and ‘strongly disagree’). The survey also captured UK region and years in the role. All responses were anonymous. The survey was written in English and was not adapted for accessibility. The steering group did not participate in the panel survey round.

Stopping criteria were agreed upon as a target of 100 responses and the threshold for consensus was set at 75% [16]. Responses were aggregated to provide an overall agreement level (i.e. the number of respondents expressing agreement as a percentage of the overall number of responses for each statement).

A results briefing was presented to the steering group at the second meeting. This information was then discussed to determine next steps and agree upon any subsequent recommendations and conclusions. If the results showed significant disagreement (e.g. >10% of statements not achieving consensus) then the steering group would revise contentious statements and a second round of survey was scheduled. The second survey contained a brief overview of the results from the first round for respondents to review prior to completion.

A statement of consent was included at the start of the survey. As this study only collected the anonymous opinions of healthcare professionals and no patient-specific data were captured, ethical approval was not sought. This study was not prospectively registered.

ACCORD (ACcurate COnsensus Reporting Document) guidelines were adhered to in presenting the findings of the current study [17].

## Results

After independent statement review, 56 statements were tested with the wider survey panel. A total of 100 completed surveys were received and all were included in the final analysis. By region, the greatest response was received from London (*n* = 24), followed by Northwest England (*n* = 21), Southeast and Southwest England (both *n* = 14), Northeast England (*n* = 12), Scotland and East Anglia (both *n* = 6), Wales (*n* = 3) and Northern Ireland (*n* = 2). A total of 19 respondents reported >20 year of experience, 39 reported 11–20 years, 23 reported 6–10 years and 19 reported up to 5 years.

**Figure 2:**
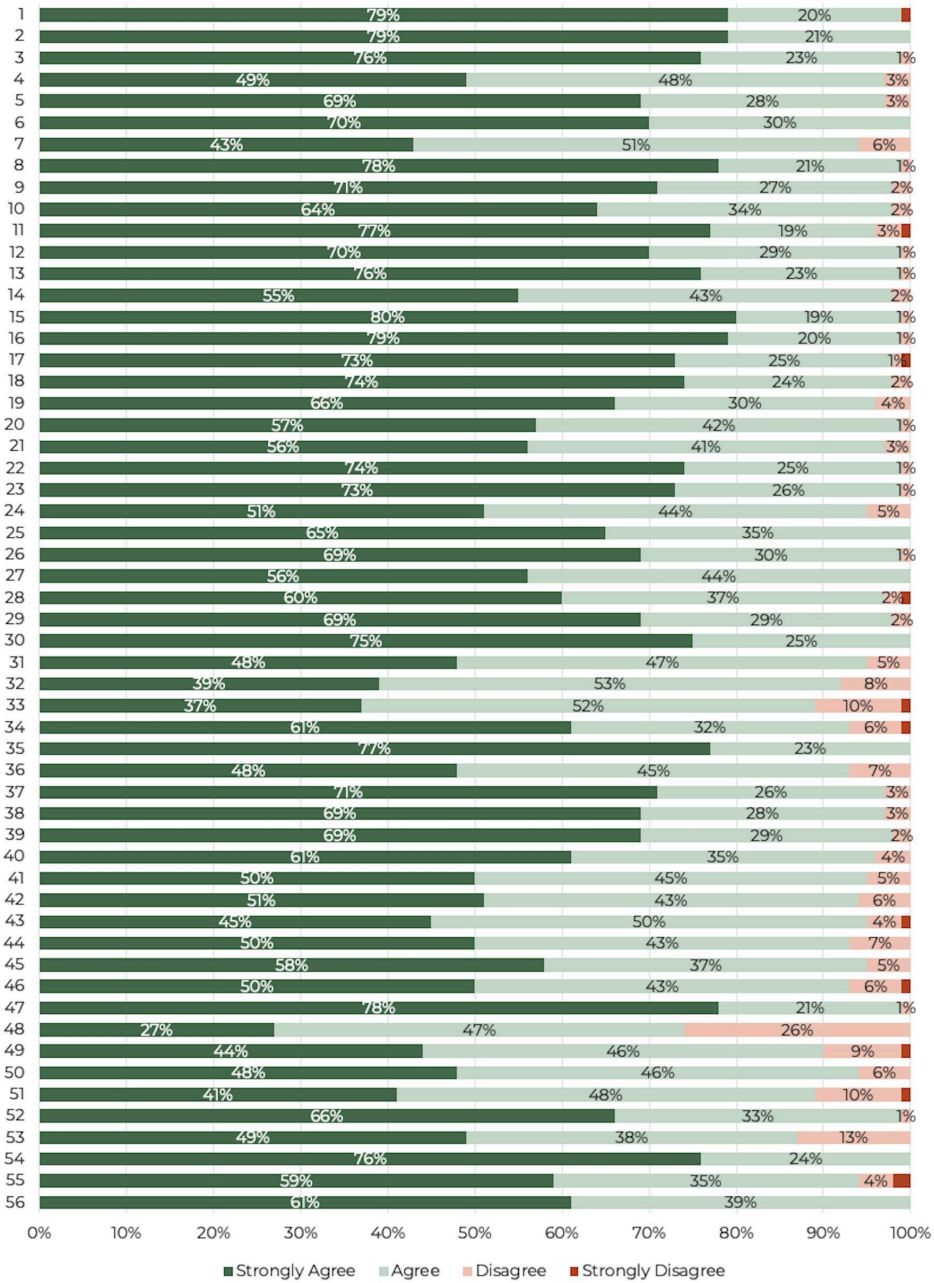
Percentage of responses by agreement level (*n* = 100)

Results demonstrated strong agreement (≥90%) in 93% (*n* = 52/56) of statements and agreement (≥75% and <90%) in 5% (*n* = 3/56) of statements. One statement (2%) narrowly failed to achieve consensus with an agreement level of 74% (Table 1, Supplementary Fig. S1 and Fig. 2). The steering group agreed that further rounds were not deemed necessary, as all statements achieved or closely approached the predefined consensus threshold (≥75%) on first testing, indicating strong alignment among respondents. Conducting further rounds was therefore unlikely to yield meaningful changes in opinion.

**Figure 3:**
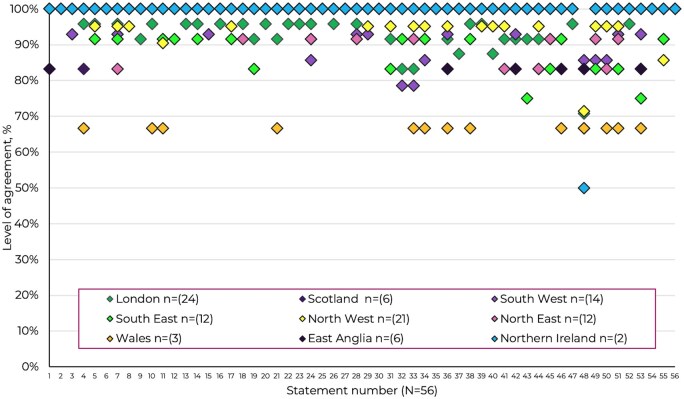
Agreement levels by region (*n* = 100)

Across regions (Fig. 3), overall agreement with the consensus statements was consistently high, typically >90%, although some regional variation was evident. Statements relating to patient empowerment, psychological support and multidisciplinary care achieved near-universal endorsement across all regions, whereas slightly lower agreement was noted in a few statements from regions with smaller response numbers—most notably East Anglia (*n* = 6) and Northern Ireland (*n* = 2). While regions with larger participation, such as London (*n* = 24) and the North West (*n* = 21), demonstrated uniformly strong agreement, lower percentages in regions with limited respondents likely reflect sample variability rather than systematic differences in opinion. Nevertheless, a small number of statements, including those concerning new care models and integrated pathways, showed more heterogeneous agreement, suggesting that implementation feasibility and service structures may differ between UK regions. Statement 48 did not achieve consensus, with an overall agreement of 74%, reflecting divergent views across regions. The highest levels of agreement were observed in the South West (86%) and North West (83%), indicating that opinions are mixed on this point. These findings indicate broad national consensus but with variations that may reflect the influence of local service capacity and sample size. Analysis of results according to respondent time in the role (Supplementary Fig. S2) did not reveal a pattern of differences between groups.

Based on these results, a set of core recommendations was developed to drive meaningful change for individuals with PsA and axSpA, with a focus on patient empowerment to improve patient outcomes and quality of life. These recommendations highlight the need for active collaboration, a supportive healthcare environment, tailored educational resources, structured measures and multidisciplinary approaches to ensure holistic, patient-centred care.

### Patient empowerment requires active participation by the patient and HCP in a permissive care environment, with support from patient education, support and advocacy organisations and individualised patient education

Strong agreement was achieved for all statements within domain A (patient empowerment in PsA and axSpA). These statements support the need for open dialogue between the patient and HCP regarding aspects of axSpA and PsA care [Statement 1 (S1), 99%; S2, 100%; S3, 99%]. Patients should be signposted to relevant patient education organisations (e.g. Versus Arthritis) to strengthen the patient voice (S4, 97%). Given the significant psychological impact of living with a chronic condition, support should be offered, including an individualised education package to support shared decision-making (S5, 97%; S6, 100%; S8, 99%). The implementation of patient-initiated follow-up (PIFU) can also support and promote patient empowerment (S12, 99%; S36, 93%).

### Specific auditable measures of patient empowerment [e.g. healthcare empowerment questionnaire (HCEQ), patient activation measures (e.g. PAM)] and patient-reported outcome measures (PROMs) should be used in the care of axSpA and PsA patients

The respondent panel supports the use of individualised patient empowerment measures (S7, 94%) that can be benchmarked and audited for service improvement. In addition, the patient activation measure (PAM) can be used to assess the degree to which patients are able to self-manage (S9, 98%) or to identify individuals who need more support in order to self-manage. To support specific and targeted discussions, the panel agreed that patients should be able to discuss their PROMs with their HCP (S10, 98%).

### Educational materials should be tailored to the individual patient to ensure opportunity of access and engagement for all, regardless of cultural and educational background

All statements in domain B (patient knowledge in PsA and axSpA) achieved strong consensus. There are a range of educational resources available to axSpA and PsA patients, but respondents agreed that recommended materials should be from reliable sources and in formats that the patient can access (S13–S18, all ≥98%). The development or selection of any patient education materials should be done in collaboration between HCPs and patients/patient education, support and advocacy organisations (S19–S21, all ≥96%). The National Axial Spondyloarthritis Society (NASS), Psoriasis and Psoriatic Arthritis Alliance (PAPAA) and Arthritis UK are useful resources for patient support materials (S22, 99%).

### Treatment goals and health priorities should be agreed upon in consultation with the patient

Respondents recognise that patients have their own motivations and priorities regarding their health that should inform the treatment approach (S24, 95%; S25, 100%; S26, 99%).

### Primary care professionals, dermatologists and allied health professionals should be educated to identify and better support axSpA and PsA

All HCPs that have touchpoints with patients require education to allow them to recognise, refer and support individuals with axSpA and PsA (S27–S30, all ≥97%).

### Treatment choices should be made based on the holistic needs of the patient, with the pharmacological choice driven by clinical need and patient-specific outcomes

Results in domain D (optimal treatment) showed that a holistic and outcome-driven approach to treatment choice (S44, 93%) is needed and cost-effectiveness alone should not drive treatment choice (S43, 95%). The presence of EMMs in PsA should be considered and treatment choices made in consultation with relevant specialists where needed (S47, 99%). Access to physiotherapy, hydrotherapy and psychological support should be available to individuals with axSpA (S54–S56, all ≥94%). Respondents also agreed that the lived experience of the patient should be listened to and considered when making treatment decisions (S38, 97%; S39, 98%). High levels of agreement across all statements in domain C (access to healthcare) demonstrate the importance of understanding each individual’s condition and goals to support appropriate, timely and tailored care.

### The patient experience should be embedded in care services

Rheumatologists agreed that axSpA and PsA patients should be managed by a multidisciplinary team (MDT) with relevant input from physiotherapists and psychologists. Flare and biologic management plans should be individualised with a contact number for prompt advice for all patients. Local clinical guidelines should be developed to reflect the clinical needs of the local patient population (S49, 90%) and the patient experience should be considered in both formulary committees and guideline development (S51, 89%). Where clinically needed, access to biologic treatments should be timely and equitable across regions and healthcare systems (S52, 99%). NHS commissioners and budgeters need to understand the patient perspective (S50, 94%) to ensure services reflect the holistic needs of the patient population and maximise the benefits of any investment.

## Discussion

**Figure 4:**
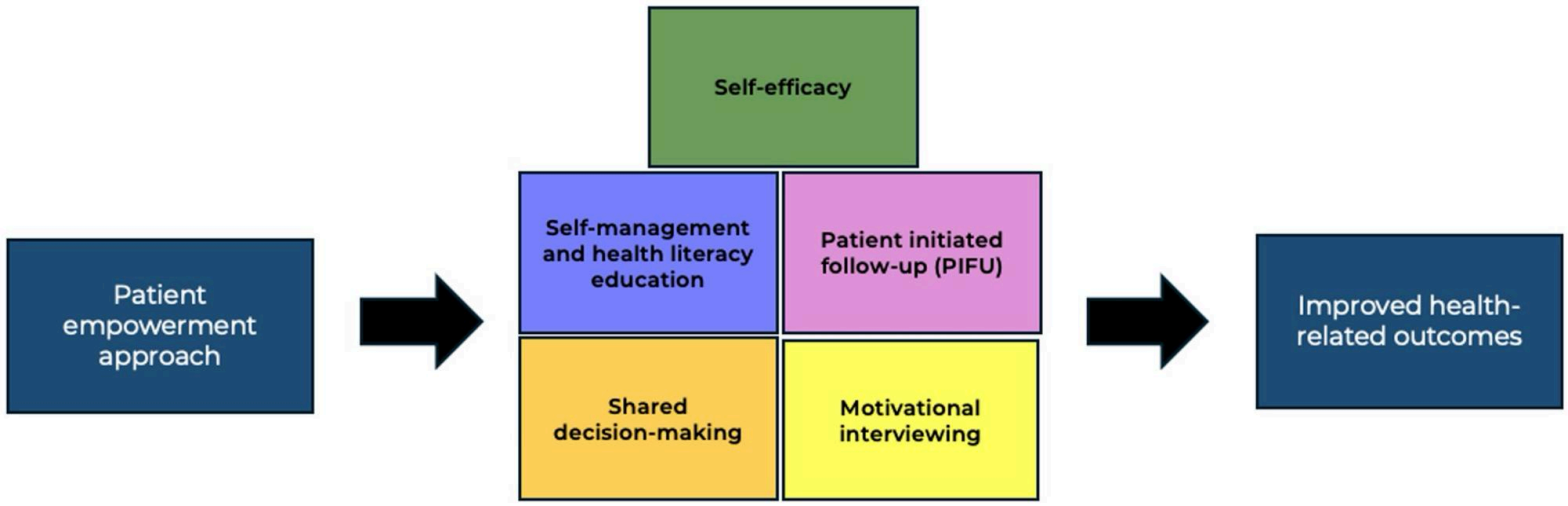
A patient empowerment approach to improve health-related outcomes in PsA and axSpA: Key building blocks identified through Delphi consensus

As depicted in Fig. 4, patient empowerment in axSpA and PsA should be underpinned by health literacy education, shared decision-making and self-efficacy and supported by strategies including motivational interviewing and PIFU to improve health-related outcomes [18–21]. This figure was developed from both existing literature and the results of this consensus.

The concept of empowering patients who live with chronic conditions is not uncommon, however, it is underutilised [22]. Empowerment approaches, and the role of self-efficacy to improve self-management, have been identified as crucial for enabling individuals to take control of, and manage, their disease [22, 23]. Self-efficacy refers to an individual’s belief in their ability to effectively handle various situations, and patients with high self-efficacy levels are more likely to positively engage in self-management activities and subsequently report improvements in health-related outcomes [24–26].

Low levels of self-management and health literacy have been linked to increased healthcare costs, faster disease progression and early mortality [27]. A Health Foundation study [28] suggests that empowering patients who currently feel least able to manage their conditions to the same level as those who feel most able could significantly reduce associated healthcare burden. This could prevent 436 000 emergency hospital admissions and 690 000 attendances at accident and emergency (A&E) departments each year [27, 28]. An independent evaluation corroborated the Health Foundation’s study findings [29]. Empowered patients with a higher level of self-management had 19% fewer general practitioner appointments and 38% fewer A&E attendances than those with lower levels of activation [29].

The primary challenge is implementing empowerment strategies with the goal of increasing self-efficacy and self-management in a resource-stretched system. The results in this study indicate that both HCPs and patients must actively participate in shared decision-making within a supportive environment. This requires prioritising patient needs and fostering a collaborative atmosphere, ideally with the support of patient education and support and advocacy organisations. This aligns with EULAR recommendations that state effective self-management requires a multipronged approach [10]. This includes educating HCPs on available strategies and resources; fostering strong collaborations between patients, patient organisations and HCPs; providing access to reliable information and implementing effective patient education programs [10]. It is imperative to recognise that knowledge alone is insufficient for successful self-management; ongoing support from a variety of sources and addressing individual needs are essential [10].

Implementing patient empowerment in real-world practice is challenging, as it is influenced by broader social determinants. Socio-economic status, health literacy, digital access and systemic healthcare inequalities all shape a patient’s ability to engage in shared decision-making and self-management [30, 31]. Efforts taken by clinicians may be limited by external factors such as regional variations in healthcare access, cultural attitudes towards self-management and disparities in support networks. A holistic, system-wide approach is required to create an environment where patient empowerment is feasible and sustainable.

Choice of treatment should be driven by individual needs and clinical outcomes, and not solely by the cost of the treatment. Experts emphasised the importance of tailoring treatment plans to individual patients, considering factors such as the patients treatment goals, disease domains and overall healthcare resource impact. Unfortunately, axSpA can be difficult to distinguish from conditions like fibromyalgia that have an overlapping symptom profile, and thus confound optimal treatment choice [32].

A 2024 Delphi consensus on elevating the SoC for patients with axSpA identified the importance of educating the patient on the reasoning behind treatment [12]. Employing techniques like motivational interviewing as a part of the patient empowerment approach promotes patient involvement in their disease management and may ultimately increase axSpA treatment adherence [33].

Educational materials should be tailored to the individual patient to ensure opportunity of access and engagement for all, regardless of cultural and educational background. Accessibility can be improved by providing language translations for those who need it and producing audible, large text or braille resources for the visually impaired. Additionally, language should be inclusive and avoid medical jargon to accommodate lower levels of health literacy. One possibility to improve the tailored education could involve adapted symptom or health-related quality of life semi-structured interviews or questionnaires [29] where the output identifies the patient’s specific area(s) of concern and signposts directly to the most appropriate resource. Subjectively, the quality and quantity of patient resources for axSpA and PsA is far from limited, but the questions of which resource or toolkit is most useful to patients and whether HCPs are aware of them remain. It may be useful in the future to survey patients with the aim of identifying and fine-tuning the top-rated resources.

Holistic treatment extends beyond immunotherapy for axSpA and PsA. Best practice should include an individualised flare management plan created collaboratively with patients. A similar effective approach exists for those with asthma [34]. A personalised asthma action plan offers individualised self-management guidance to help patients maintain asthma control during stable periods and regain control during exacerbations [34]. An axSpA or PsA flare plan should include a point of contact, such as the clinic helpline, and an existing patient resource on flare management, such as the ‘Managing my axial SpA (AS) flares’ booklet from the NASS [35].

Allied health professionals within the multidisciplinary team can play a key role in patient empowerment. Nurses, in particular, significantly contribute to chronic disease management by providing essential care and educating patients about their conditions [24]. Evidence indicates that nurse-led care in RA, using a person-centred approach and strong communication skills, creates a positive therapeutic environment by providing psychological and educational support that empowers patients [36]. Also, for RA, web-based interventions to support patient education and empowerment show great success [37]. The Patient Empowerment to Medication Adherence Programme (PE2MAP) for RA is an effective and importantly feasible supportive asset [37]. Up to 92% (*n* = 24) and 88% (*n* = 23) of individuals agreed that the program improved their confidence in discussing treatments with their HCPs and improved their adherence to medications and knowledge on managing side effects, respectively [37]. There is an opportunity to adapt the PE2MAP for those with axSpA and PsA.

PIFU is also regarded as a key part of patient empowerment within rheumatology [38, 39]. PIFU empowers patients and caregivers by providing them with the autonomy to schedule follow-up appointments according to their specific symptoms and circumstances rather than adhering to a rigid timetable [38, 39]. This independence aims to reduce unnecessary hospital outpatient appointments, thereby relieving appointment backlog, which is worsened by an increasing workforce shortfall [38, 39]. PIFU has demonstrated an equivalent safety and efficacy profile compared with traditional care models across multiple therapy areas [39]. Adopting digital tools, such as electronic patient reported outcome measures (ePROMs) and the Health Care Empowerment Questionnaire (HCEQ) [40] to facilitate PIFU, shows promise as a safe and specific method of active remote monitoring and streamlining escalation of patient care [39].

In the 2024/25 NHS England priorities and operational and planning guidance, all providers and systems must remove 65-week waits by September 2024 (except where patients have elected to wait longer or in specific specialties) [41]. Moreover, systems are tasked with increasing the number of PIFUs by 4.5% *vs* the 2022/23 targets [41]. Including PIFUs in the patient empowerment strategy should be prioritised for axSpA and PsA care.

While wider adoption of PIFU and integration of ePROMs are consistent with NHS England’s digital self-management agenda, their implementation must account for existing workforce and infrastructure constraints. Many departments already operate at or beyond capacity, and extended follow-up intervals leave limited flexibility to release additional clinical time. In this context, PIFU and PROM systems should not be viewed as additional workload but as mechanisms to triage patients more effectively—ensuring that clinic time is directed toward those with the greatest need. Automated ePROM alerts and remote review dashboards, integrated into existing electronic health record systems, could support monitoring without requiring extra appointments or administrative burden. Over time, the efficiencies gained through better-targeted reviews and reduced unnecessary follow-ups may offset the initial implementation effort, but this will require investment in digital infrastructure, staff training and equitable access to technology.

This consensus aligns with the Pan-European Rheumacensus ‘calls to action’ [12], which identified patient empowerment, education, optimal treatment and enhanced HCP–patient communication as central priorities for improving standards of care in PsA and axSpA. While both emphasise the importance of shared decision-making, multidisciplinary collaboration and accessible patient education, this consensus builds on these by contextualising them within the structure and policy environment of the NHS. Specifically, it addresses local implementation challenges such as regional variations in access, commissioning through ICSs and the drive toward digital self-management and waiting-time reduction.

There is also alignment with ongoing NHS England priorities aimed at improving patient autonomy and reducing system pressures through digital and self-management initiatives, including NHS England’s digital self-management agenda, which seeks to enhance remote monitoring and empower patients to manage chronic conditions outside traditional clinical settings [42]. Furthermore, embedding these strategies within rheumatology pathways could contribute to achieving national waiting-time reduction targets by streamlining follow-up care and optimising clinic capacity [41].

Although digital tools such as ePROMs and PIFU offer opportunities to enhance empowerment and efficiency, implementation may be constrained by digital inequalities across the UK. Limited internet access in rural areas, variable digital literacy and age-related barriers can reduce engagement with online platforms. Hybrid approaches tailored to the local population that combine digital and face-to-face support are therefore needed.

However, there is concern that to some patients the concept of self-management and patient empowerment may lead them to believe that patients must cope with their condition alone [10]. Also, some patients may prefer to not take an active role in decision-making and prefer to be told what to do regarding their treatment [10]. Consequently, it is important to assess an individual’s health literacy and tailor approaches accordingly on a case-by-case basis [43].

### Next steps

**Figure 5:**
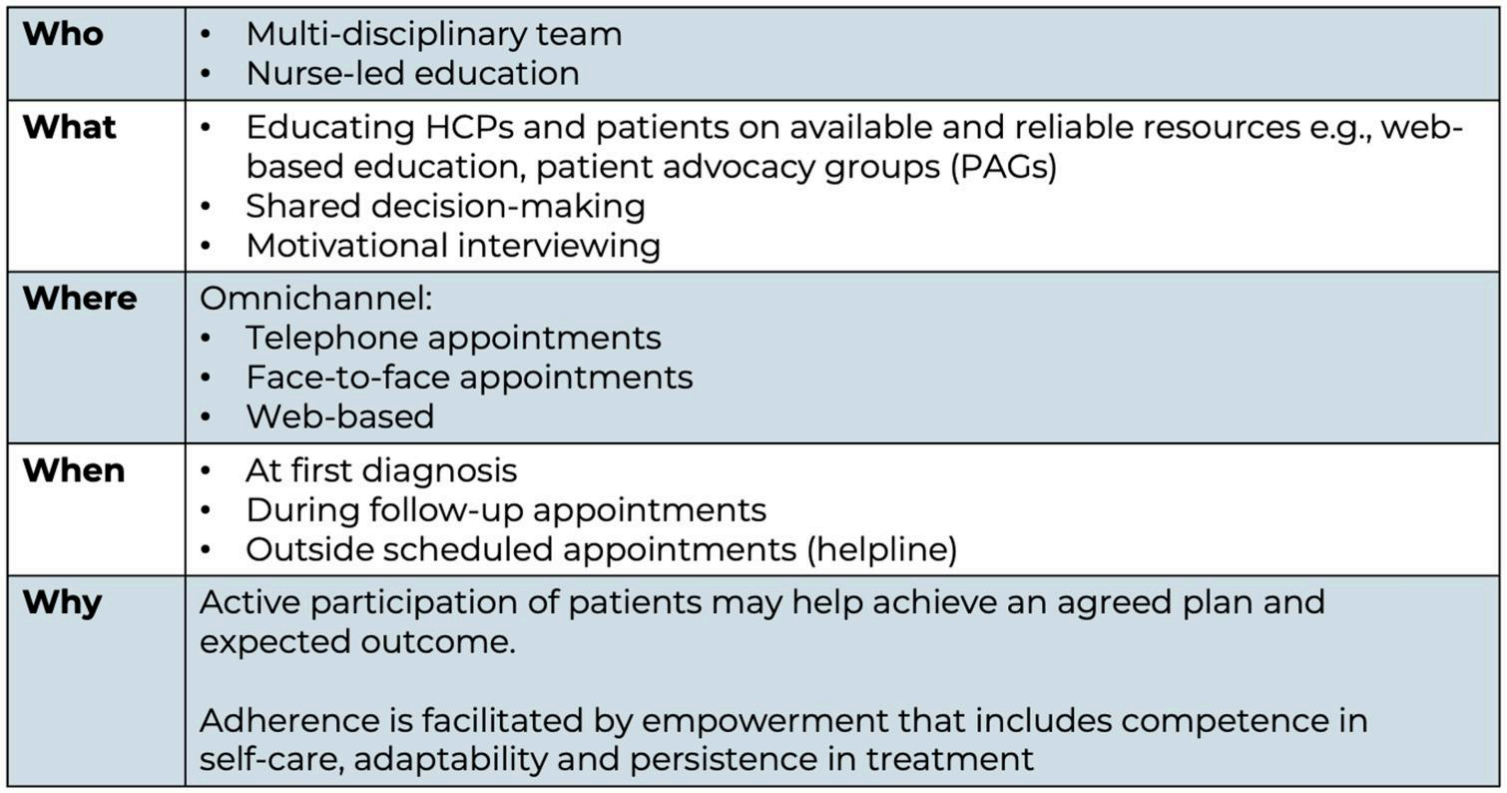
Who, what, where, when and why: a framework for patient engagement and empowerment in PsA and axSpA care

The framework presented in Fig. 5 was developed using steering group discussions and existing evidence. It outlines the key components required to embed patient empowerment and collaborative care within UK rheumatology practice. The next phase should focus on translating these principles into measurable service improvements through the co-development of implementation toolkits, audit standards and educational resources for multidisciplinary teams. Collaboration between professional societies (e.g. BSR), patient organisations (e.g. NASS, PAPAA, Arthritis UK) and NHS commissioners will be essential to ensure these recommendations are operationalised across ICSs. Future work should also evaluate the impact of these strategies—such as PIFU, PROMs integration, and tailored educational interventions—on clinical outcomes, patient activation and system efficiency. By aligning this framework with EULAR’s international self-management and empowerment agenda [10], these efforts can help position the UK as a model for implementing patient-centred care.

### Limitations

There was strong agreement for all but one of the consensus statements, which narrowly failed to reach the predefined threshold. Such high agreement may suggest that the statements were not provocative enough to challenge current thinking, but the purpose of this consensus was to establish agreement on what best practice should look like. While the steering group reviewed the statements for accuracy and suitability, the statements may have benefitted by being piloted prior to the initial survey. The respondent rheumatologists clearly agreed with the proposed statements, which suggests that there is a need to better understand how these practices can become standard practice embedded in the NHS.

The study focused on a select group of consultant rheumatologists’ views and did not include other disciplines typically involved in axSpA and PsA multidisciplinary teams or patient feedback. The experience and opinions of patients is vital to improving provision for chronic conditions. This consensus is a first step in aligning PsA and axSpA care more closely with the needs of patients; further work is planned, including the potential ratification of these findings with a panel of patient representatives [44]. While the survey respondents demonstrate a broad geographic distribution across the UK, the highest representation was from experts in London and North West England, with participation from Wales and Northern Ireland notably lower. This may limit the generalisability of findings due to potential differences in patient demographics and existing healthcare infrastructure and resources. Additionally, there is a slight weighting towards more experienced rheumatologists, which could result in established clinical perspectives being favoured over emerging viewpoints. Future studies may benefit from quota-driven recruitment strategies to ensure a fully representative national perspective.

## Conclusion

The strong consensus achieved confirms alignment from experts that best practice care for patients with axSpA and PsA should prioritise patient empowerment, with the aim of improving health outcomes and quality of life. A holistic approach that includes patient education, shared decision-making and self-management strategies can significantly enhance patient experiences and reduce the burden on healthcare systems. By implementing patient-centred care models, such as PIFU and the use of digital education tools, healthcare providers can empower patients to take control of their chronic condition. However, it is essential to tailor these approaches to individual patient needs and preferences, considering factors like health literacy and personal circumstances. Further research is needed to evaluate the quality of resources available to signpost patients and HCPs.

Patient engagement in axSpA and PsA is a shared responsibility across the MDT at every point of patient contact and through all communication channels. Each touchpoint should support patient empowerment through educational resources, motivational interviewing and shared decision-making, all of which can enhance adherence and improve outcomes [2, 11, 45].

## Supplementary Material

rkag005_Supplementary_Data

## Data Availability

Anonymised data are available upon request.
