# Supplementary material for: SpArking change for patients with psoriatic arthritis and axial spondyloarthritis in the UK: results from a UK Delphi consensus study
Source: Rheumatol Adv Pract. 2026 Jan 9;10(1):rkag005. doi: 10.1093/rap/rkag005 (PMC12857574; doi:10.1093/rap/rkag005)
Supplement: rkag005_Supplementary_Data [file rkag005_supplementary_data.docx]

# Supplementary materials

# **Supplementary Figure S1: Overall agreement levels (n=100)**

**
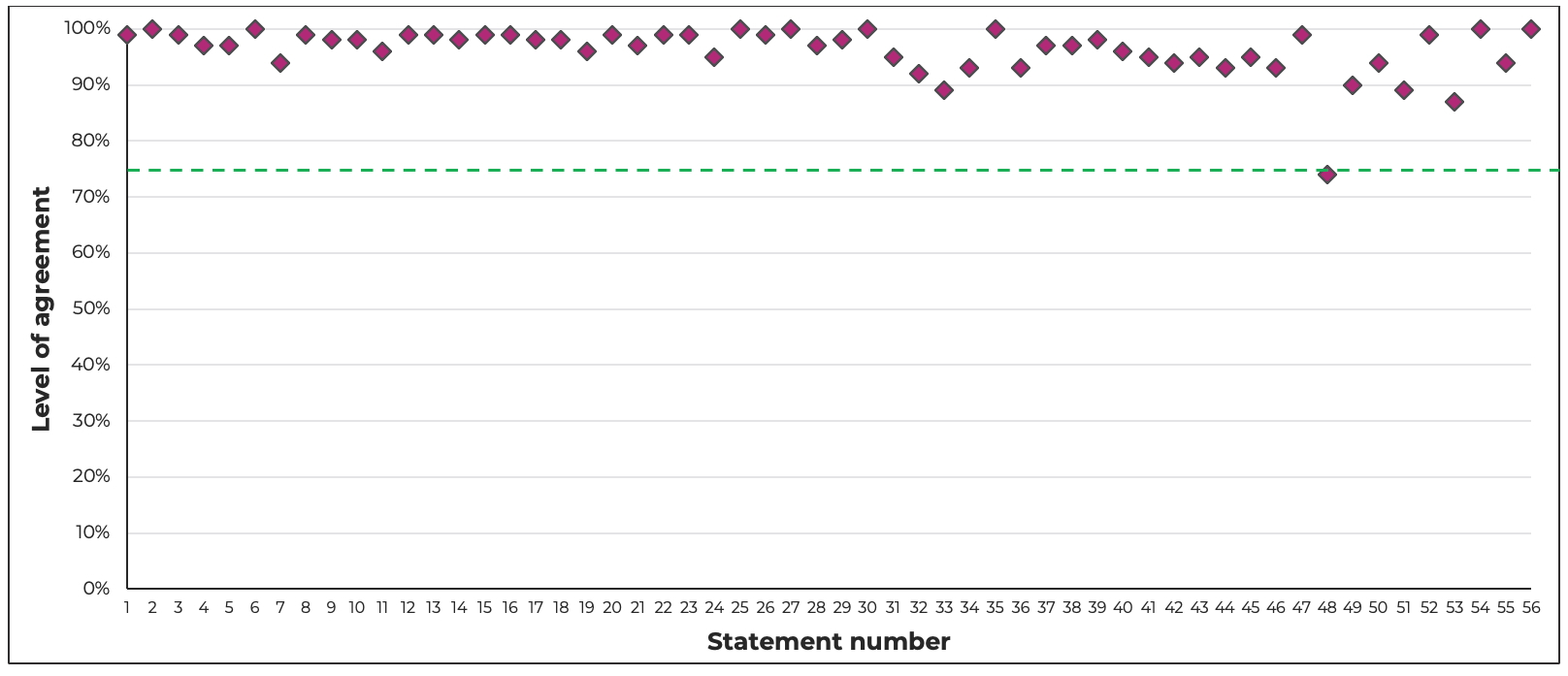
** The green line indicates the threshold for consensus agreement (75%)

**Supplementary Figure S2: Agreement levels by time in role (n=100)**
